# The incidence of diabetes mellitus and its determining factors in a Kurdish population: insights from a cohort study in western Iran

**DOI:** 10.1038/s41598-024-66795-3

**Published:** 2024-07-09

**Authors:** Farid Najafi, Mehdi Moradinazar, Fatemeh Khosravi Shadmani, Yahya Pasdar, Mitra Darbandi, Yahya Salimi, Seyed Ramin Ghasemi

**Affiliations:** 1 Research Center for Environmental Determinants of Health (RCEDH), Health Institute, Kermanshah University of Medical Sciences, Kermanshah, Iran; 2 Social Development and Health Promotion Research Center, Health Institute, Kermanshah University of Medical Sciences, Kermanshah, Iran; 3 Student Research Committee, Department of Epidemiology, School of Public Health, Kermanshah University of Medical Sciences, Kermanshah, Iran

**Keywords:** Diabetes mellitus, Incidence, Cohort, RaNCD, Iran, Diseases, Medical research, Risk factors

## Abstract

Diabetes mellitus (DM) is among the most widespread non-communicable diseases and poses a substantial global health challenge. The aim of this study was to examine the incidence of DM and its nutritional, anthropometric, laboratory, demographic, and behavioral determinants, as well as comorbidities, within a Kurdish population residing in western Iran. This research was conducted in the Ravansar Non-Communicable Disease (RaNCD) cohort study, followed 9170 participants aged 35–65 years, for an average ± SD of 7.11 ± 1.26 years, from 2015 until 2023. A hierarchical Cox regression model was used to estimates the adjusted hazard ratios (HRs). The incidence of DM was 4.45 (95% CI 3.96, 4.99) per 1000 person-years. We found several significant predictors for DM incidence, including prediabetes, comorbidity, urban residence, total antioxidant capacity (TAC), and the interaction between gender and body mass index (BMI). Prediabetes emerged as the strongest predictor of DM incidence, with a hazard ratio of 10.13 (CI 7.84, 13.09). Additionally, having two diseases (HR = 2.18; 95% CI 1.44, 3.29) or three and more diseases (HR = 3.17; 95% CI 2.06, 4.90) increased the risk of developing DM. Also, the hazard ratios for the effects of gender on DM incidence in the normal, overweight, and obese BMI groups were 0.24, 0.81, and 1.01, respectively. The presence of prediabetes and obesity serve as the crucial indicators for the onset of DM, emphasizing the pressing need for interventions to prevent DM in these circumstances. Furthermore, there are notable disparities between urban and rural populations in this study, warranting further investigations to ascertain the underlying causes of such variations.

## Introduction

Non communicable diseases (NCDs) are the main cause of death worldwide^1,2^. Diabetes mellitus (DM) is one of the most common NCDs^3^ and is a significant global health challenge. It has emerged as one of the fastest-growing health emergencies^4^, with its incidence doubling between 1980 and 2014^5^. In 2021, the global prevalence of diabetes was 6.1%, affecting approximately 529 million individuals. This number is projected to more than double to 1.3 billion people within the next 30 years^6^. It is also estimated that in 2021, diabetes-related causes accounted for approximately 6.7 million deaths, 90% of which were attributed to DM^7,8^. Between 1990 and 2021, the prevalence of diabetes in the North Africa and the Middle East regions increased by 161.5%, surpassing the global rate by 71%^6^. In Iran, based on the Persian cohort study, the sex- and age-standardized prevalence of diabetes and prediabetes among 35–75 year-olds were found to be 15.0% and 25.4%, respectively^9^. Additionally, according to statistics from the International Diabetes Federation, the estimated prevalence of diabetes among the adult population in Iran in 2021 was 9.5%^10^. These statistics show that despite all the valuable efforts at the global and national levels in combating this disease, there appears to be no evidence of a reduction in the increasing trend^11,12^.

Diabetes leads to numerous complications and limitations for patients^13,14^. Moreover, poorly controlled diabetes can significantly increase the risk of various health issues, including atrial fibrillation^15^, heart failure and peripheral arterial disease^16^, aortic valve stenosis^17^ and periodontal diseases^18^. Additionally, diabetes diminishes quality of life, affects functional capacity, and contributes to morbidity and premature mortality^11^. The DM, in particular, is associated with a wide range of different risk factors^19^ that interact in a complex manner, increasing the difficulty of understanding its incidence formation. Therefore, monitoring the incidence and prevalence of diabetes holds immense importance from various prospective, including prevention and treatment, and can provide valuable benefits for the health system^20^.

Diabetes is a chronic disease^6^ influenced by various factors. Older age^8,21^, female sex^22^ and high BMI^6,23^ are among the major risk factors for the development of DM. Despite extensive research in this field, it remains crucial to gain a better understanding of disparities in risk factor profiles and the burden of diabetes across different populations^6^. Furthermore, there is a clear need for studies to be conducted in diverse societies to address this gap. Considering the significant socioeconomic changes in Iran in recent years, potential shifts in the incidence of DM and its determinants are expected. While some studies have examined diabetes incidence in Iran, such as that of Najafipour et al. in Kerman, Southeast Iran, who reported an incidence rate of 1.2 per 100 person-years for DM during a 5-year follow-up period^23^, and that of Ebrahimi et al. in Shahroud, who found a 5-year incidence of diabetes of 13.73%^24^, no study has been found on the incidence of diabetes in the western region of Iran, particularly among the Kurdish ethnic group, both within Iran and globally. Therefore, this is the first a prospective cohort study in western Iran, specifically among the Kurdish population, to investigate the incidence of DM and its determining factors and to provide valuable insights into this disease. It would enable a better understanding of DM and facilitate more effective measures to address its spread, particularly in this geographical area.

## Methods

### Study population

**Figure 1:**
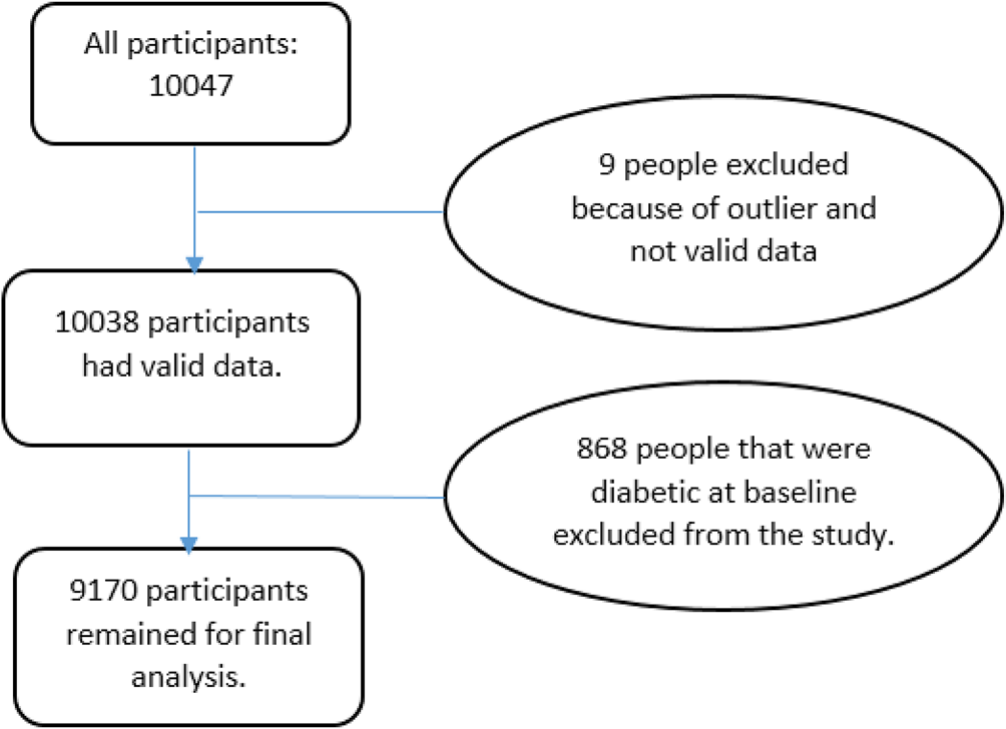
The flowchart of people who participated in the study (also 572 participants have been censored follow-up duration: 234 death; 329 withdrawal; 9 migration).

The Ravansar Non-Communicable Disease (RaNCD) cohort study is one of the 18 subsets of Prospective Epidemiological Research Studies in Iran (the main or adult) Prospective Epidemiological Research Studies in Iran (PERSIAN) Cohort Study. In the RaNCD cohort study, 10,047 participants aged 35–65 years were followed up for 7.04 ± 1.40 years (range: 0.003–8.52 years), and their general, medical, and nutritional characteristics were recorded. Details of the study have been published previously^25^. The Ravansar County is located in the west of the Kermanshah province, Iran; in close proximity to the country of Iraq. Most of the residents of this city are Kurdish. Additionally, complete information about this cohort is available on the Persian cohort website^26^ and relevant published papers^27^. The present study was approved by the research ethics committee of Kermanshah University of Medical Sciences (Ethics code: IR.KUMS.REC.1402.332); Also all the participants provided oral and written informed consent. This research was conducted on all RaNCD cohort study participants (10,047) that followed from 2015 to 2023. After excluding 9 participants because of outlier data and 868 participants because of DM history at baseline, 9170 individuals (4796 women, 4374 men) were included in the analysis (Fig. 1). The maximum missing values for the baseline data variables were 115 (1.14%) (for example, BMI: 115; fasting blood sugar (FBS): 63; healthy eating index (HEI): 44 missing values) which were replaced by the multiple imputation method. In addition, during the follow up, 234, 329 and 9 participants (572 in total) were censored because of death, withdrawal and migration, respectively.

### Covariate measurements

The baseline assessment was performed between November 12, 2014, and December 31, 2015. The data were collected by trained study personnel using a standardized protocol. Risk factors for DM incidence were included in this study based on the literature review and available data from the RaNCD cohort study. The demographic and socioeconomic variables included socioeconomic status (SES), age, gender, marital status, education years, residence type and job status. Lifestyle information in three categories included behavioral variables (smoking status, sleep duration, alcohol use and metabolic equivalent of task (MET)), nutritional variables (using the Iranian Food Frequency Questionnaire including percent carbohydrate kcal, percent fat kcal, percent protein kcal, energy, Dietary Inflammatory Index (DII), Healthy Eating Index (HEI), plant diet index score (PDI), total antioxidant capacity quantiles (TAC)) and anthropometric variables (BMI, waist circumference (WC), waist-hip ratio (WHR), body fat mass(BFM), skeletal muscle mass (SMM)and percent body fat (PBF)) were measured. The laboratory variables included fasting blood sugar (FBS), cholesterol, triglyceride levels, low- and high-density lipoprotein cholesterol (LDL-C and HDL-C) levels,, serum BUN, serum creatinine, the aspartate aminotransferase (SGOT), the alanine aminotransferase (SGPT), Alkaline phosphatase (ALP) and Gamma-glutamyl trans-peptidase (GGT). Detailed information about the data characteristics can be found in the RaNCD cohort profile article^25^.

### Diabetes prevalence

Diabetes prevalence was defined for those, who had a fasting plasma glucose (FPG) level of $\ge$ 126 mg/dl, and/or were on diabetes medication and/or had diabetes confirmed by a health practitioner at recruitment phase. The prevalent cases were eliminated from analysis. The p*rediabetes variable* was constructed using FBS variable in such a way that a person with FBS ≤ 99.99 was considered as “No Diabetes/ Prediabetes”; 100 ≤ FBS ≤ 125.99 as “Prediabetes” and FBS ≥ 126 as Diabetes. *Dyslipidemia* was considered to be LDL cholesterol ≥ 160 mg/dl and/or total cholesterol ≥ 240 mg/dl and/or HDL cholesterol < 40 mg/dl and/or triglycerides ≥ 200 mg/dl and/or a history of taking medications for dyslipidemia. *Comorbidity* was defined based on a history of 7 diseases, namely, hypertension, cancer, renal failure, fatty liver, thyroid disease (Self-report), cardiovascular disease (CVD), and depression.

### Follow-up measurements

During the follow-up phase, all participants were interviewed annually by phone, as well as after the occurrence of an event of interest. The follow-up was conducted using both active and passive methods. For the annual follow-up, each participant was contacted via telephone, or if needed, they were seen by a physician at the study center. In addition to active follow-up, passive follow-up involved the collection of self-reports from participants whenever they visited the center to report an event occurrence. Annual reports from disease and death registries were also obtained^25^.

### Outcome variable

During the annual follow-up of all participants, those with suspected signs and symptoms are actively referred to medical doctors outside the cohort center for further evaluation and diagnosis of diabetes. Such participants, as well as those who have been diagnosed by their medical doctor during routine medical visits, are followed for the results of their medical investigations. All medical records (including prescriptions of anti-hyperglycemic agents) will be collected by a general physician and then reviewed by at least two out of three physicians (internists) at the Outcome Review Committee for approval of the diabetes diagnosis (ICD-10-CM: E11). The diagnostic criteria for diabetes mellitus (DM) are based on the standards defined by the American Diabetes Association (ADA). Participants are considered to have diabetes if they meet at least two of the following laboratory criteria:

- Fasting plasma glucose level of [≥ 126 mg/dL (7.0 mmol/L)]
- Hemoglobin A1c (HbA1c) level of [≥ 6.5% (48 mmol/mol)]
- 2-h plasma glucose level of [≥ 200 mg/dL (11.1 mmol/L)] during a 75-g oral glucose tolerance test (OGTT).
- Or are currently prescribed one or more antidiabetic medications by a healthcare provider.

In cases where the two reviewing physicians disagree on the diagnosis, a third reviewing physician (also an internist) provides the final diagnosis^25^.

### Statistical analysis

Descriptive statistics (mean, standard deviation, or frequency, percent) were used to analyze the baseline data of the study participants. Additionally, the overall prevalence of the main covariates at the baseline level was reported. Differences in baseline characteristics between diabetes incidence/non-incident groups were examined by Student’s t-test for comparisons of means and chi-square test and Fisher exact test for comparisons of proportions. Cox proportional hazard models were used to calculate hazard ratios (HRs) and 95% confidence intervals (CIs) for incident diabetes associated with various risk factors. To achieve this, three Cox models were executed. Model 1 included demographic, behavioral, anthropometric and nutritional covariates, namely, age, gender, residency, marital status, alcohol and smoking status, BMI, percent-protein-kcal, TAC, HEI, and metabolic equivalent of task (MET). Model 2 was expanded on Model 1 by including disease profile variables, namely, comorbidity, dyslipidemia and pre-diabetes, in addition to the variables from Model 1. Finally, Model 3 included interaction term, “Gender* BMI”, alongside the variables from Model 2. Covariates with a p-value less than 0.2 were included in the multivariate Cox model. Furthermore, certain variables were excluded from the models due to collinearity. The Schoenfeld residuals test (both global and scaled) was used to check the proportionality assumption of hazard estimation. Firth's Cox regression was also conducted to examine potential bias resulting from data imbalance. However, since the results obtained from Firth's Cox regression closely resembled those obtained from the conventional PH Cox method, only the findings from later method were presented. All analyses were conducted with Stata 17 and R 3.6.3 softwares and p < 0.05 was considered to indicate statistical significance.

### Ethics approval and consent to participate

We confirm that all methods related to the human participants were performed in accordance with the Declaration of Helsinki and approved by Research Ethics Committee of Kermanshah University of Medical Sciences. This study also received ethics approval from the Research Ethics Committee of Kermanshah University of Medical Sciences (No.IR.KUMS.REC.1402.332).

## Results

From 10,047 participants, 9170 eligible persons were investigated and followed for 7.11 ± 1.26 years on average (range: 0.003–8.52 years). The mean ± SD age of all participants was 46.91 ± 8.23 years, and 4796 (52.30%) were women.

The incidence of diabetes was 4.45 cases per 1000 person-years (95% CI 3.96, 4.99). Specifically, the incidence rate was 5.22 per 1000 person-years (95% CI 4.50, 6.04) for women and 3.62 per 1000 person-years (95% CI 3.01, 4.35) for men. The total number of incident cases was 290 (177 cases (61.03%) women) and their mean age was 49.50 ± 7.77 years (Female: (n = 177): 49.76 ± 8.20 years; Male (n = 113): 49.08 ± 7.77 years). Other findings showed that 42.41% of the incident cases were nonsmokers, while 8.28% were current smokers. Also, there were statistically significant differences between incident cases and none-diabetic group in terms of age, education years, gender, residence type, job status, marital status, smoking status and BMI (p < 0.05). The distributions of the other variables between the two groups are reported in Table 1.

**Table 1:** Distribution of baseline characteristics of participants based on diabetes status.

			Diabetes incidence (n = 290)	No diabetes (n = 8880)	Total (n = 9170)	*p*
			Mean ± SD n (%)	Mean ± SD n (%)	Mean ± SD n (%)	
Demographic variables	Age		49.50 ± 7.77	46.82 ± 8.24	46.91 ± 8.23	< 0.001
	Gender	Female	177 (61.03%)	4619 (52.02%)	4796 (52.30%)	0.002
	Residence type	Urban	205 (70.69%)	5220 (58.78%)	5425 (59.16%)	< 0.001
	Marital status	Single	2 (0.69%)	408 (4.59%)	410 (4.47%)	0.003
		Married	265 (91.38%)	7998 (90.07%)	8263 (90.11)	
		Divorced	19 (6.55%)	359 (4.04%)	378 (4.12)	
		widowed	4 (1.38%)	115 (1.30%)	119 (1.30)	
	SES quantiles	Poorest	56 (19.31%)	1795 (20.21%)	1851 (20.19%)	0.715
		2	54 (18.62%)	1760 (19.82%)	1814 (19.78%)	
		3	56 (19.31%)	1771 (19.94%)	1827 (19.92%)	
		4	67 (23.10%)	1747 (19.67%)	1814 (19.78%)	
		Richest	57 (19.66%)	1807 (20.35%)	1864 (20.33%)	
Behavioral variables	Sleep duration (hour per 24 h)		7.08 ± 1.30	7.11 ± 1.21	7.11 ± 1.22	0.729
	Metabolic equivalent of task (MET/hour per day)		40.00 ± 8.30	41.17 ± 8.30	41.13 ± 8.31	0.035
	Alcohol use	Yes	9 (3.10%)	443 (4.99%)	452 (4.93%)	0.017
	Smoking status	Not smoking	123 (42.41%)	3674 (41.37%)	3797 (41.41%)	0.125
		Current smoker	24 (8.28%)	1039 (11.70%)	1063 (11.59%)	
		Former smoker	32 (11.03%)	725 (8.16%)	757 (7.26%)	
		Passive smoker	111 (38.28%)	3442 (38.76%)	3553 (38.75%)	
Nutritional variables	Carbohydrate (%E)		60.87 ± 6.33	61.48 ± 6.16	61.46 ± 6.17	0.098
	Fat (%E)		27.28 ± 6.02	26.79 ± 5.92	26.80 ± 5.93	0.160
	Protein (%E)		13.96 ± 2.36	13.69 ± 2.16	13.70 ± 2.16	0.038
	Energy intake (kcal/day)		2724.44 ± 984.48	2711.55 ± 968.02	2711.96 ± 968.49	0.823
	Dietary inflammatory index (DII)		− 2.24 ± 1.56	− 2.35 ± 1.59	− 2.34 ± 1.59	0.273
	Healthy eating index (HEI)		52.54 ± 7.27	51.50 ± 7.33	51.53 ± 7.33	0.017
	Plant diet index score (PDI)		54.54 ± 6.80	54.30 ± 6.66	54.31 ± 6.66	0.557
	Total antioxidant capacity quantiles (TAC)	1	56 (19.31%)	2235 (25.17%)	2291 (24.98%)	0.062
		2	79 (27.24%)	2220 (25.00%)	2299 (25.07%)	
		3	69 (23.79%)	2227 (25.08%)	2296 (25.04%)	
		4	86 (29.66%)	2198 (24.75%)	2284 (24.91%)	

Concerning the incidence of diabetes based on the years of follow-up, the numbers of individuals diagnosed with diabetes after 1–9 years of observation were 36, 47, 51, 37, 26, 44, 33, 13, and 3, respectively.

Regarding the status of underlying diseases at baseline, 72.41%, 28.97% and 25.86% of incident cases had fatty liver disease, cardiovascular disease and high blood pressure, respectively. In terms of BMI, 8.62% of incident cases were normal and underweight and 47.59% were obese. Additionally, 56.55% and 68.28% of the incident cases had dyslipidemia and prediabetes, respectively (Table 2).

**Table 2:** Distribution of baseline characteristics of participants based on diabetes status.

			Diabetes incidence (n = 290)	No diabetes (n = 8880)	Total (n = 9170)	*p*
			Mean ± SD n (%)	Mean ± SD n (%)	Mean ± SD n (%)	
Laboratory variables	BUN		13.66 ± 3.99	13.54 ± 4.16	13.55 ± 4.15	0.636
	Creatinine		0.98 ± 0.21	0.99 ± 0.24	0.99 ± 0.23	0.321
	SGOT (The aspartate aminotransferase)		22.73 ± 9.25	21.43 ± 8.97	21.47 ± 8.98	0.015
	SGPT (The alanine aminotransferase)		29.52 ± 17.11	24.42 ± 14.54	24.58 ± 14.65	< 0.001
	ALP (Alkaline phosphatase)		209.74 ± 112.37	195.23 ± 59.08	195.69 ± 61.52	0.001
	GGT (Gamma-glutamyl trans-peptidase)		32.40 ± 23.02	23.58 ± 17.79	23.86 ± 18.99	< 0.001
	Dyslipidemia	Yes	164 (56.55%)	3696 (41.62%)	3860 (42.09%)	< 0.001
	Prediabetes	Yes	198 (68.28%)	1184 (13.33%)	1382 (15.07%)	< 0.001
Comorbidity	Cancer*	Yes	5 (1.72%)	64 (0.72%)	69 (0.75%)	0.052
	Renal failure	Yes	41 (14.14%)	910 (10.25%)	951 (10.37%)	0.032
	Hypertension	Yes	75 (25.86%)	1218 (13.72%)	1293 (14.10%)	< 0.001
	Fatty liver	Yes	210 (72.41%)	3175 (35.75%)	3385 (36.91%)	< 0.001
	Thyroid disease*	Yes	38 (13.10%)	646 (7.27%)	684 (7.46%)	< 0.001
	CVD	Yes	84 (28.97%)	1249 (14.07%)	1333 (14.54%)	< 0.001
	Depression	Yes	17 (5.86%)	269 (3.03%)	286 (3.12%)	0.006
Anthropometric variables	Waist circumference (WC)		102.67 ± 9.78	96.80 ± 10.50	96.99 ± 10.53	< 0.001
	Waist hip ratio (WHR)		0.97 ± 0.06	0.94 ± 0.06	0.94 ± 0.06	< 0.001
	Body fat mass (BFM)		30.62 ± 9.21	24.65 + 9.48	24.84 ± 9.53	< 0.001
	Skeletal muscle mass (SMM)		26.86 ± 5.64	26.43 ± 5.75	26.44 ± 5.75	0.214
	Percent body fat (PBF)		38.37 ± 7.99	33.47 ± 9.53	33.62 ± 9.53	< 0.001
	Body mass index (BMI)	Normal and underweight	25 (08.62%)	2832 (31.89%)	2857 (31.16%)	< 0.001
		Overweight	127 (43.79%)	3835 (43.19%)	3962 (43.21%)	
		Obese	138 (47.59%)	2213 (24.92%)	2351 (25.63%)	
Time to event/censoring (year)			3.63 ± 2.14	7.22 ± 1.04	7.11 ± 1.26	< 0.001

*Self report.

**Figure 2:**
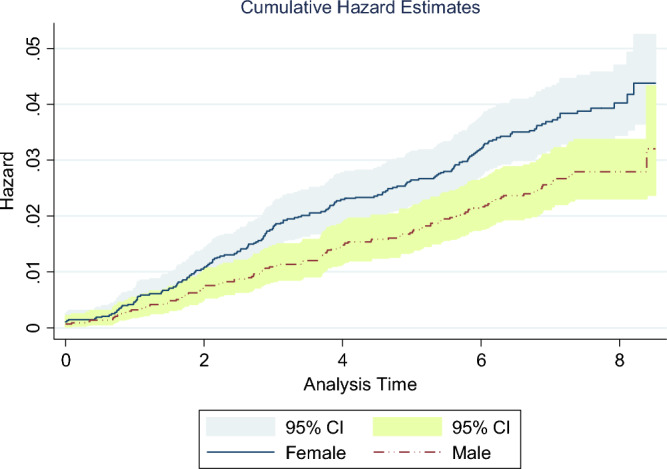
Nelson–Aalen cumulative hazard for type 2 diabetes among male and female participants (log-rank test, P = 0.002).

The cumulative hazard of DM among male and female participants during the study period is shown in Fig. 2. There were 177 incident DM cases among 4796 females and 113 incident DM cases among 4374 male participants. The cumulative hazard (DM incidence) was consistently lower among males than among females and this difference was significant (log-rank test, *P* = 0.002).

**Figure 3:**
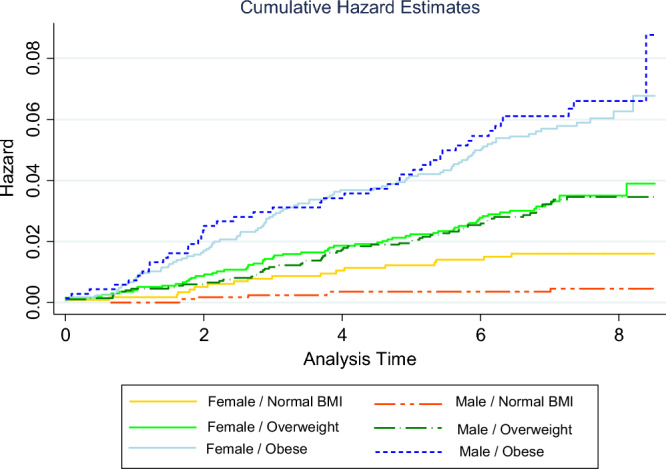
Nelson–Aalen cumulative hazard for type 2 diabetes across different gender and BMI groups (log-rank test, P ≤ 0.001).

The hazard ratios for the effects of sex on diabetes incidence in the normal, overweight, and obese BMI groups were 0.24, 0.81, and 1.01, respectively. These findings indicate that the incidence of diabetes is estimated to be 76% and 19% lower in males than in females in the normal and overweight BMI groups, respectively. However, there is an estimated 1% greater incidence of diabetes in males than in females in the obese BMI group. Figure 3 depicts the cumulative hazard for type 2 diabetes across different gender and BMI groups.

Based on Cox proportional hazards analysis in Model 3 (full model), while controlling for the impact of other covariates, gender, residence type, TAC, comorbidity, prediabetes and the interaction term of Gender*BMI emerged as significant predictors of diabetes incidence. Notably, prediabetes exhibited the strongest predictive power for diabetes incidence, with a hazard ratio of 10.22 (95% confidence interval: 7.91–13.21). Also, the results of Model-3 showed a significant main effect of gender on diabetes incidence (HR = 0.24; CI 0.10–0.60), indicating that the incidence of diabetes is greater in females than in males. However, the effect of BMI on diabetes incidence was not significant. The Schoenfeld residual global test confirmed that the overall full model satisfied the assumption of proportional hazards (chi-squared = 24.48, df = 25, p-value: 0.492). The results of the survival analysis are presented in Table 3.

**Table 3:** The results of Cox regression analysis of the determinants of diabetes incidence (n = 9170).

Variables	Crude model HR (95% CI)	Adjusted model-1^a^ HR (95% CI)	Adjusted model-2^b^ HR (95% CI)	Adjusted model-3^c^ HR (95% CI)
Age	1.04 (**1.02–1.05**)	1.04 (**1.03–1.06**)	1.00 (0.98–1.02)	1.00 (0.98–1.02)
Gender^1^				
Male	0.69 (**0.55–0.88**)	0.83 (0.62–1.10)	0.81 (0.61–1.09)	0.24 (**0.10–0.60**)
Residence type^2^				
Urban	1.51 (**1.17–1.95**)	1.28 (0.97–1.68)	1.51 (**1.14–2.00**)	1.52(**1.15–2.02**)
Marital status^3^				
Married	6.30 (**1.57–25.34**)	3.46 (0.85–14.05)	3.02 (0.74–12.26)	3.23 (0.79–13.15)
Divorced	10.09 (**2.35–43.32**)	3.78 (086–16.58)	3.48 (0.79–15.29)	3.67 (0.83–16.15)
Widowed	6.94 (**1.27–37.91**)	4.36 (0.79–23.95)	4.71 (0.67–20.51)	4.08 (0.74–22.57)
Alcohol use^4^				
Yes	0.61 (0.31–1.18)	0.75 (0.38–1.51)	0.67 (0.34–1.35)	0.67 (0.33–1.35)
Smoking status^5^				
Current smoker	0.70 (0.45–1.09)	1.04 (0.65–1.67)	1.14 (0.71–1.83)	1.17 (0.73–1.89)
Former smoker	1.33 (0.90–1.96)	1.26 (0.84–1.91)	1.16 (0.77–1.76)	1.15 (0.76–1.73)
Passive smoker	0.97 (0.75–1.25)	0.96 (0.74–1.24)	0.90 (0.70–1.17)	0.91 (0.70–1.18)
Metabolic equivalent of task (MET)	0.98 (0.96–1.00)	0.99 (0.98–1.01)	1.00 (0.98–1.02)	1.00 (0.99–1.02)
Protein (%E)	1.05 (**1.00–1.11**)	1.07 (**1.01–1.13**)	1.05 (0.99–1.11)	1.05 (0.99–1.11)
Sleep duration	0.98 (0.89–1.08)	–	–	–
Dietary inflammatory index	1.02 (0.95–1.09)	–	–	–
Healthy eating index (HEI)	1.01 (**1.00–1.03**)	0.99 (0.97–1.01)	0.99 (0.97–1.01)	0.99 (0.97–1.01)
Total antioxidant capacity quantiles (TACq)^6^				
2	1.37 (0.98–1.93)	1.37 (0.97–1.95)	1.48 (**1.04–2.09**)	1.48 (**1.04–2.10**)
3	1.19 (0.83–1.69)	1.19 (0.82–1.72)	1.27 (0.87–1.84)	1.28 (0.88–1.87)
4	1.46 (**1.04–2.05**)	1.44 (0.99–2.10)	1.52 (**1.03–2.23**)	1.52 (**1.03–2.23**)
Dyslipidemia^7^				
Yes	1.79 (**1.42–2.26**)	–	1.20 (0.94–1.53)	1.18 (0.92–1.50)
Pre–diabetes^8^				
Yes	13.28 (**10.37–17.02**)	–	10.17 (**7.87–13.14**)	10.22 (**7.9–13.21**)
Comorbidity^9^				
One disease	2.90 (**2.06–4.07**)	–	1.41 (0.97–2.05)	1.34 (0.92–1.94)
Two disease	4.99 (**3.45–7.23**)	–	2.24 (**1.48–3.40**)	2.18 (**1.44–3.29**)
Three disease and more	8.13 (**5.64–11.71**)	–	3.28 (**2.12–5.07**)	3.17 (**2.06–4.90**)
Body mass index (BMI)^10^				
Overweight	3.65 (**2.37–5.60**)	3.52 (**2.29–5.43**)	2.05 (**1.31–3.20**)	1.25 (0.73–2.12)
Obese	6.69 (**4.37–10.25**)	6.21 (**4.00–9.63**)	2.46 (**1.53–3.96**)	1.42 (0.84–2.43)
Interaction (gender*BMI)	–	–	–	
Male # overweight	–	–	–	3.39 (**1.31–8.73**)
Male # obese	–	–	–	4.21 (**1.62–10.92**)

^a^Model with demographic, behavioral, anthropometric and nutritional covariates include age, gender, residency, marital status, alcohol and smoking status, BMI, percent-protein-kcal, TACG, HEI, and metabolic equivalent of task (MET) (Model AIC: 5098.07).

^b^Variables of model-1 plus disease profile include comorbidity, dyslipidemia and pre-diabetes covariates (Model AIC: 4704.27).

^c^Variables of model-2 plus the interaction term Gender* BMI (Model AIC: 4698.58).

Reference groups:1-Female; 2-Rural; 3-Single; 4-No alcohol use; 5-Not-smokers; 6-First quantile; 7-No dyslipidemia; 8-No pre-diabetes; 9-No disease; 10-underweight and normal.

Significant values are given in bold.

**Figure 4:**
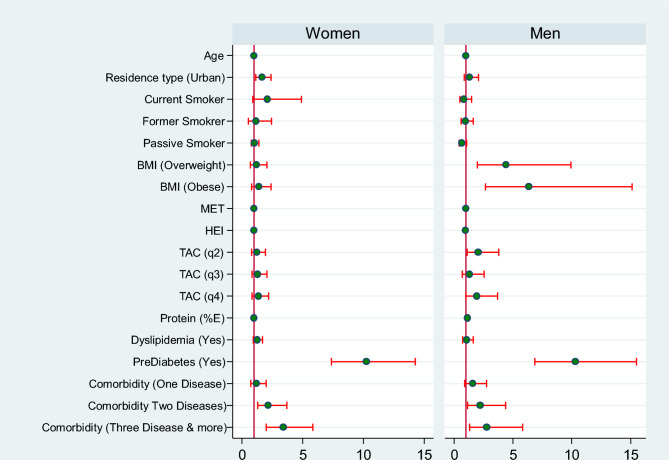
Forest plot of HRs (95% CIs) of determining factors of DM across males and females.

Also, we implemented the final model (model-3) separately for women and men. Figure 4 depicts Forest plot of HRs (95% CIs) of Determining Factors of DM across males and females. The graph illustrates that, apart from the BMI variable, the results for other covariates are almost similar in both men and women.

## Discussion

This study investigated the incidence of diabetes and its determinants through a prospective cohort study. Our study revealed an incidence rate of 4.45 per 1000 person-years for DM. Additionally, we identified several influential factors, including prediabetes, comorbidity, urban residence, and the interaction between gender and BMI.

Previous studies have reported varying diabetes incidence rates across different societies. Sharma et al. in a study in Mumbai, India reported an incidence rate of 5.3 cases per 1000 person-years^28^. In a study conducted in Korea, Hyun et al. reported a higher incidence rate of 22.8 per 1000 person-years for DM^22^. Similarly, Rojo-Martínez et al. observed an adjusted overall incidence rate of 11.6 per 1000 person-years in Spain^29^. Zhang et al. reported an incidence rate of 10.0 per 1000 person-years in their study^21^. In Iran, Najafipour et al. reported an incidence rate of 12 per 1000 person-years for DM^23^. In total, diabetes incidence varies based on multiple factors, such as population characteristics such as ethnicity^30^, lifestyle^31^, healthcare systems ability^32^, and genetic predisposition^33,34^. Therefore, our study demonstrated lower incidence rates than did these aforementioned studies.

According to our fully adjusted model, prediabetes emerged as the strongest predictor of diabetes incidence. A hazard ratio of 10.22 was associated with a history of prediabetes, indicating a tenfold greater risk of developing diabetes in participants with prediabetes than in those without prediabetes. This finding is consistent with previous studies, although the effect sizes in our study were greater. For example, Wang et al. reported a hazard ratio of 2.35 for diabetes incidence among individuals with baseline prediabetes in their study^35^.

Furthermore, our results indicated a consistently lower cumulative hazard for DM incidence among males than females. Similarly, Ghafouri et al., in a cross sectional study among rural residents of Kurdistan Province, Iran, reported a significantly greater prevalence of type 2 diabetes in women^36^. In contrast, Zhang et al. found a lower incidence of diabetes in women than in men, and these associations held even after adjusting for confounding factors^21^. However, the findings from the comprehensive model, taking into account the interaction between gender and BMI, revealed that the impact of gender on the occurrence of diabetes varied across different categories of BMI. Specifically, among individuals with a BMI within the normal and overweight range, women exhibited a greater incidence of diabetes than men did. In contrast, among individuals classified as obese, men displayed a slightly greater (1%) risk of developing diabetes than women did. This finding indicates that the influence of gender on the risk of developing diabetes varies among different BMI categories. These interactions can be explained by various factors, including hormonal^37,38^, genetic^38,39^, and lifestyle factors, as well as by the divergence in body fat distribution between males and females^40^. For instance, the differences in the distribution of body fat between genders may contribute to the divergent diabetes risk observed across distinct BMI categories. However, further research is needed to determine the underlying factors contributing to these gender-specific differences in diabetes risk according to BMI categories.

However, while the second model showed that BMI had a significant role in the incidence of diabetes, with hazard ratios of 2.05 and 2.46 for overweight and obese individuals, respectively, compared to the normal group; this significant effect vanished after considering its interaction with gender. This finding suggested that the impact of BMI on diabetes risk is not solely dependent on BMI alone, but it is also influenced by an individual's gender. The interaction between BMI and gender indicates that the relationship between BMI and diabetes incidence varies depending on whether an individual is male or female. This interaction suggests that there might be underlying gender-specific factors that modify the relationship between BMI and diabetes risk. In contrast to our study, Najafipour et al. reported a direct relationship between diabetes incidence and BMI^23^. Also, Logue et al. showed that men have a lower BMI around the time of diagnosis of diabetes than women do^37^.

Comorbidity was identified as a significant determinant of DM in this study. The risk of diabetes incidence was greater in individuals with "two" or "three or more" underlying diseases, with hazard ratios of 2.18 and 3.17, respectively, than in those without any comorbidities. Notably, this increased risk does not establish a causal relationship. Instead, these findings underscore the importance of individuals with preexisting conditions taking additional measures to safeguard their health. The presence of these underlying conditions amplifies the probability of developing diabetes in such individuals, thereby emphasizing the necessity for intensified health management.

In our study, we found that the variable of place of residence was a significant determinant of diabetes incidence. Specifically, the hazard of developing diabetes was observed to be 1.52 times greater in urban residents than in their rural counterparts. Similarly, Zhao et al. reported that diabetes was more prevalent among urban older adults than their rural counterparts in Southwest China^41^. The reasons behind this disparity in diabetes risk based on place of residence can be multifactorial. Compared with rural regions, urban areas often exhibit distinct characteristics such as higher population density, increased access to processed foods, sedentary lifestyles, and limited opportunities for physical activity. Nonetheless, the present study adjusted for variables such as physical activity and the Healthy Eating Index (HEI) in the regression model.

### Strength and limitations

One of the strengths of this research lies in its distinctive focus on investigating the incidence of diabetes and its determinants within the Kurdish population, utilizing a cohort study design and a substantial sample size. Moreover, the study demonstrated notable success in mitigating loss to follow-up, with a mere 5.7% attrition rate. Nevertheless, it is crucial to acknowledge the limitations inherent in this study. The generalizability of the findings to other population subgroups may be limited because the specific population under investigation was confined to the west of Iran. Furthermore, despite meticulous efforts to control for confounding factors, the potential for unmeasured or residual confounding cannot be entirely ruled out.

Notably, this study represents the first investigation into the incidence of DM in the western region of Iran and within the Kurdish regions worldwide, among 9170 adults with a mean follow-up duration of 7.11 years. Our findings revealed important insights into the burden of diabetes in this population. Additionally, our analysis identified several determining factors that were associated with the development of diabetes among the population. These findings contribute to the existing literature by shedding light on the unique epidemiological characteristics of diabetes within this population and providing valuable information for public health strategies and interventions tailored to the society.

## Conclusion

The results revealed that prediabetes status, comorbidity and urban residence, are the most important independent factors influencing the incidence of DM. In addition, while males are less likely to develop diabetes, obese males are more likely to develop diabetes indicating a positive interaction between gender and BMI. Therefore, it is strongly recommended to implement targeted interventions within this population group to minimize the burden of diabetes in the foreseeable future. Additionally, it is crucial to focus on monitoring BMI differences between genders, as well as between urban residents, through an effective surveillance system.

## Data Availability

The data sets used and analyzed in this study are available from the corresponding author on reasonable request.

## Abbreviations

RaNCD: Ravansar non-communicable disease
HR: Hazard ratio
CI: Confidence intervals
DM: Diabetes mellitus
BMI: Body mass index
TAC: Total antioxidant capacity
NCDs: Non communicable diseases
FBS: Fasting blood sugar
HEI: Healthy eating index
SES: Socioeconomic status
MET: Metabolic equivalent of task
DII: Dietary Inflammatory Index
PDI: Plant diet index score
BUN: Blood urea nitrogen
SGOT: The aspartate aminotransferase
SGPT: The alanine aminotransferase
ALP: Alkaline phosphatase
GGT: Gamma-glutamyl trans-peptidase
FPG: Fasting plasma glucose
LDL-C: Low density lipoprotein cholesterol
HDL-C: High density lipoprotein cholesterol
WC: Waist circumference
WHR: Waist-hip ratio
BFM: Body fat mass
SMM: Skeletal muscle mass
PBF: Percent body fat
CVD: Cardiovascular disease
